# TRPM4 links calcium signaling to membrane potential in pancreatic acinar cells

**DOI:** 10.1016/j.jbc.2021.101015

**Published:** 2021-07-27

**Authors:** Gyula Diszházi, Zsuzsanna É. Magyar, Erika Lisztes, Edit Tóth-Molnár, Péter P. Nánási, Rudi Vennekens, Balázs I. Tóth, János Almássy

**Affiliations:** 1 Department of Physiology, Faculty of Medicine, University of Debrecen, Debrecen, Hungary; 2 Department of Ophthalmology, University of Szeged, Szeged, Hungary; 3 Laboratory of Ion Channel Research, Department of Cellular and Molecular Medicine, Faculty of Medicine, TRP Research Platform Leuven, VIB Center for Brain and Disease Research, KU Leuven, Leuven, Belgium

**Keywords:** pancreas, acinar cells, calcium signaling, calcium imaging, calcium entry, ion channel, transient receptor potential channels, TRPM4, physiology, patch clamp, 9-ph, 9-phenanthrol, [Ca^2+^]_i_, intracellular Ca^2+^ concentration, CBA, 4-chloro-2-[[2-(2-chlorophenoxy)acetyl]amino]benzoic acid, CPA, cyclopiazonic acid, DMEM, Dulbecco's modified Eagle's medium, ER, endoplasmic reticulum, IP_3_, inositol 1,4,5-trisphosphate, IP_3_R, IP_3_ receptor, ITPR, inositol 1,4,5-trisphosphate receptor, PAC, pancreatic acinar cell, PMCA, plasma membrane Ca^2+^ ATPase, qPCR, quantitative PCR, RyR, ryanodine receptor, SERCA, sarco-ER Ca^2+^ ATPase, SOCE, store-operated Ca^2+^ entry, TRPC3, transient receptor potential canonical 3, TRPM4, transient receptor potential cation channel subfamily M member 4, TRPM5, transient receptor potential cation channel subfamily M member 5

## Abstract

Transient receptor potential cation channel subfamily M member 4 (TRPM4) is a Ca^2+^-activated nonselective cation channel that mediates membrane depolarization. Although, a current with the hallmarks of a TRPM4-mediated current has been previously reported in pancreatic acinar cells (PACs), the role of TRPM4 in the regulation of acinar cell function has not yet been explored. In the present study, we identify this TRPM4 current and describe its role in context of Ca^2+^ signaling of PACs using pharmacological tools and TRPM4-deficient mice. We found a significant Ca^2+^-activated cation current in PACs that was sensitive to the TRPM4 inhibitors 9-phenanthrol and 4-chloro-2-[[2-(2-chlorophenoxy)acetyl]amino]benzoic acid (CBA). We demonstrated that the CBA-sensitive current was responsible for a Ca^2+^-dependent depolarization of PACs from a resting membrane potential of −44.4 ± 2.9 to −27.7 ± 3 mV. Furthermore, we showed that Ca^2+^ influx was higher in the TRPM4 KO- and CBA-treated PACs than in control cells. As hormone-induced repetitive Ca^2+^ transients partially rely on Ca^2+^ influx in PACs, the role of TRPM4 was also assessed on Ca^2+^ oscillations elicited by physiologically relevant concentrations of the cholecystokinin analog cerulein. These data show that the amplitude of Ca^2+^ signals was significantly higher in TRPM4 KO than in control PACs. Our results suggest that PACs are depolarized by TRPM4 currents to an extent that results in a significant reduction of the inward driving force for Ca^2+^. In conclusion, TRPM4 links intracellular Ca^2+^ signaling to membrane potential as a negative feedback regulator of Ca^2+^ entry in PACs.

Pancreatic acinar cells (PACs) are the major cell types of the exocrine pancreas. They are responsible for secretion of digestive enzymes and primary fluid. Stimulation by endogenous secretagogues, such as acetylcholine and cholecystokinin, causes inositol 1,4,5-trisphosphate (IP_3_) generation, and consequent Ca^2+^ release from the endoplasmic reticulum (ER) through the IP_3_ receptor (IP_3_R) Ca^2+^ channels. The subsequent increase in intracellular Ca^2+^ concentration ([Ca^2+^]_i_) triggers the exocytosis of digestive enzymes (1, 2, 3, 4). During this process, termed stimulus–secretion coupling, changing [Ca^2+^]_i_ may exhibit various spatiotemporal patterns, depending on the magnitude of secretagogue stimulation, which eventually determines the quality and quantity of secretion. Threshold concentrations of secretagogues induce transient and repetitive elevations (oscillations) of [Ca^2+^]_i_, highly localized to the apical pole of PAC, which was demonstrated to elicit exocytosis of enzyme containing vesicles (5, 6, 7, 8, 9). The spatial limitation of Ca^2+^ release was explained by the higher density of IP_3_Rs in this region and the large Ca^2+^ buffering capacity of a mitochondrial belt surrounding the apical area (10, 11). Higher secretagogue concentrations cause higher [Ca^2+^]_i_ that breaks through the mitochondrial firewall and generates propagating Ca^2+^ waves, which initiate transepithelial fluid secretion as well (12, 13, 14). These patterns of Ca^2+^ signals represent the physiological function of Ca^2+^ signaling, whereas unduly high concentrations of secretagogues initiate a pathological chain of reactions, beginning with an initial [Ca^2+^]_i_ peak, followed by a lower, but sustained Ca^2+^ plateau (9, 15). These, peak–plateau-type signals overload the cell with excess amount of Ca^2+^, which is enough to trigger intra-acinar zymogen activation, self-digestion, leading to acute pancreatitis (16, 17, 18). However, both long-lasting oscillatory- and peak–plateau-type Ca^2+^ signals require Ca^2+^ influx from the extracellular environment (19, 20, 21, 22). The mechanism for Ca^2+^ entry may be either store independent or store-operated Ca^2+^ entry ([SOCE] or capacitative Ca^2+^ entry) (23, 24, 25, 26). The trigger for SOCE is the significant depletion of the ER Ca^2+^ content, and its role is to fuel further Ca^2+^ release during strong stimulation. Otherwise, either type of Ca^2+^ entry channels are assembled from different isoforms of the ORAI protein, with possible contribution of transient receptor potential canonical 3 (TRPC3) channels to SOCE (27, 28, 29).

Following [Ca^2+^]_i_ elevation, Ca^2+^ is pumped out from the cytosol by the plasma membrane Ca^2+^ ATPase (PMCA) or transferred back to the ER by the sarco-ER Ca^2+^ ATPase (SERCA) (30, 31, 32, 33).

Since the spatiotemporal characteristics of Ca^2+^ signaling fundamentally determine cell behavior and disordered Ca^2+^ signaling is directly linked to pancreatic pathology, the major challenge of research in this field is to learn more about the regulation of [Ca^2+^]_i_ and to find new pharmacological targets and compounds to prevent Ca^2+^ overload (34, 35). In our efforts to find new effectors of Ca^2+^ signaling in PACs, we examined whether Ca^2+^-regulated ion channels from the transient receptor potential family (transient receptor potential cation channel subfamily M member 4 [TRPM4] and transient receptor potential cation channel subfamily M member 5 [TRPM5]) are expressed in PACs and whether they affect Ca^2+^ signaling. First, we performed a comprehensive quantitative PCR (qPCR) analysis using murine pancreas and got a positive result for the TRPM4.

TRPM4 is an [Ca^2+^]_i_-activated nonselective cation channel mediating a significant amount of depolarizing current in several cell types (36). Accordingly, plasma membrane depolarization because of TRPM4 activation was demonstrated to control various physiological processes through the activation of voltage-gated Ca^2+^ channels (in breath pacemaker neurons and cerebral arterioles) or by decreasing capacitative Ca^2+^ entry by limiting the electrochemical driving force for Ca^2+^ influx (in mast cells and T-lymphocytes) (37, 38, 39, 40). A cation current with the hallmarks of TRPM4 was also reported in PAC by Maruyama and Petersen in 1982 (41, 42); however, studying the role of the current in PAC function was impeded by the lack of pharmacological and genetic tools at that time. Nevertheless, the cation current was suggested to be responsible for the Ca^2+^-dependent transepithelial Na^+^ and water transport required for fluid secretion. Importantly, the fact that the current is controlled by extracellular Ca^2^ implies that it may serve as a negative feedback regulator of Ca^2+^ influx. Presuming that the inward cation current was carried by TRPM4, its role in the feedback regulation of Ca^2+^ influx was tested in this study.

## Results

**Figure 1:**
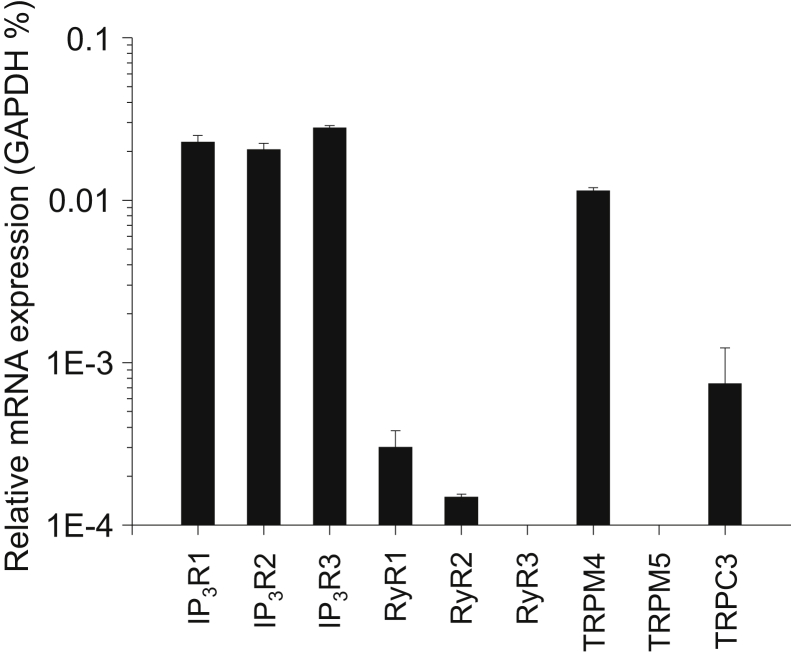
**Relative expression of ion channel genes in murine pancreas.** mRNA expression of the ion channels indicated were determined using quantitative real-time PCR. Transcripts of GAPDH were used as internal control. The assay included three replicates.

In preliminary experiments, RT-qPCR analysis was performed from murine whole pancreas lysates using DNA primer probes designed to recognize the two types of Ca^2+^-dependent cation channels TRPM4 and TRPM5. Parallel experiments were done using primer pairs against the three isoforms of IP_3_R, the three isoforms of the ryanodine receptor (RyR) Ca^2+^ release channel and TRPC3, which served as internal control. The housekeeping gene GAPDH expression was used as reference (Fig. 1). IP_3_R isoforms showed high and identical expression, whereas RyR expression was relatively low, with RyR1 being the major isoform. These results are in accordance with previous results showing that all IP_3_R isoforms are equally highly expressed and that RyR has only a complementary role in the Ca^2+^ signaling of PACs (13, 14, 43, 44, 45). TRPM4 expression was comparable to IP_3_R expression and was significantly higher than that of TRPC3, the Ca^2+^ permeable channel partially responsible for SOCE in PACs (29). TRPM5 expression level fell below the detection threshold. As TRPM5 has been shown to be highly expressed in the endocrine pancreas (46), our negative result also implies that mRNA contamination of our whole pancreas lysate by Langerhans islets did not bias our data. Therefore, we conclude that TRPM4 mRNA is highly expressed in PAC.

**Figure 2:**
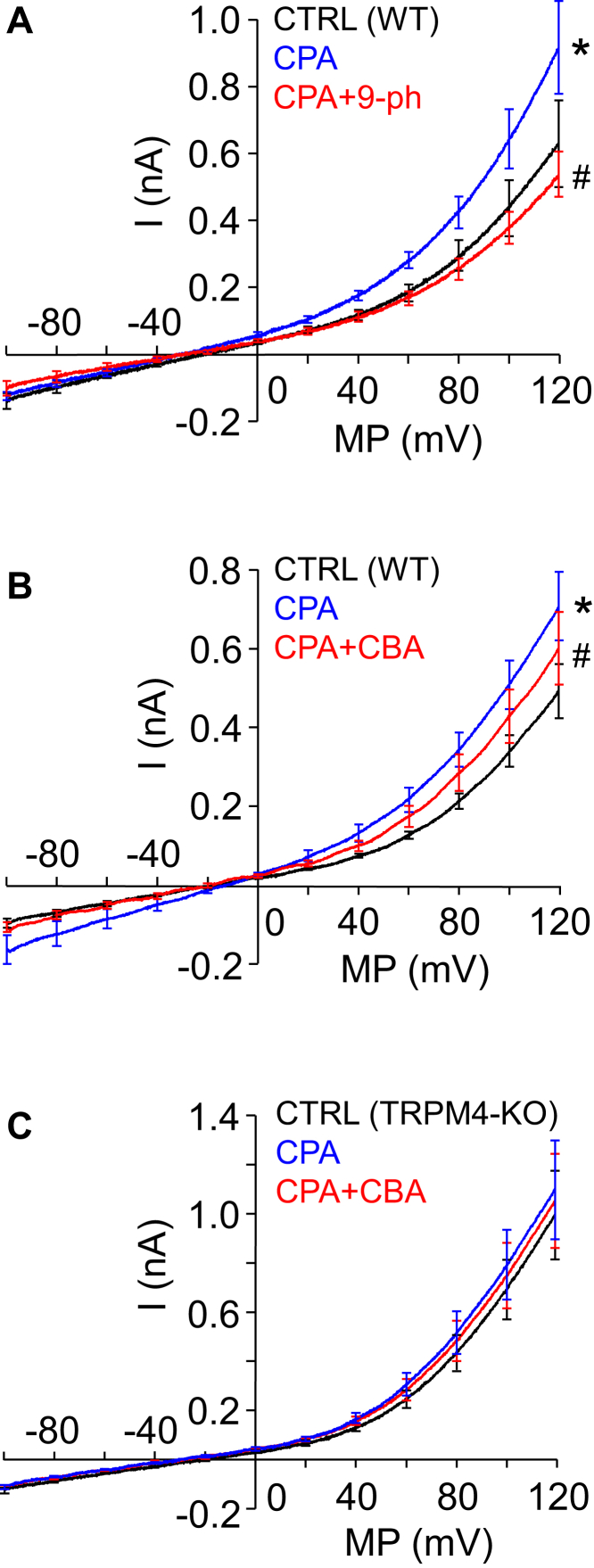
**Biophysical and pharmacological properties of the Ca**^**2+**^**-activated cation current in mouse pancreatic acinar cells.** Average of whole-cell current recordings obtained using the patch clamp technique with a ramp voltage protocol. Measurements were performed on single pancreatic acinar cells or small clusters of 2 to 3 cells isolated from WT (*A* and *B*) or TRPM4-KO (*C*) mice. Most of Cl^−^ was omitted from the recording solutions, and K^+^ was substituted with Cs^+^ in order to selectively measure Na^+^ and Cs^+^ currents of TRP channels. Ca^2+^-dependent currents were elicited using the Ca^2+^ mobilizer cyclopiazonic acid (CPA). Average of currents under control conditions (*black line*, CTRL), in the presence of 30 μM CPA (*blue line*) and during the application of TRPM4 inhibitors 9-phenanthrol (100 μM) and CBA (10 μM) along with CPA (*red line*, CPA + CBA) are displayed. (*A*, n = 7; *B*, n = 5; and *C*, n = 6). Mean currents measured at 120 mV were compared with repeated-measures ANOVA, and pairwise comparisons between the indicated groups were carried out using paired-sample *t* tests with Bonferroni correction. *Asterisks* indicate significant (*p* < 0.05) differences. CBA, 4-chloro-2-[[2-(2-chlorophenoxy)acetyl]amino]benzoic acid; TRPM4, transient receptor potential cation channel subfamily M member 4.

**Figure 3:**
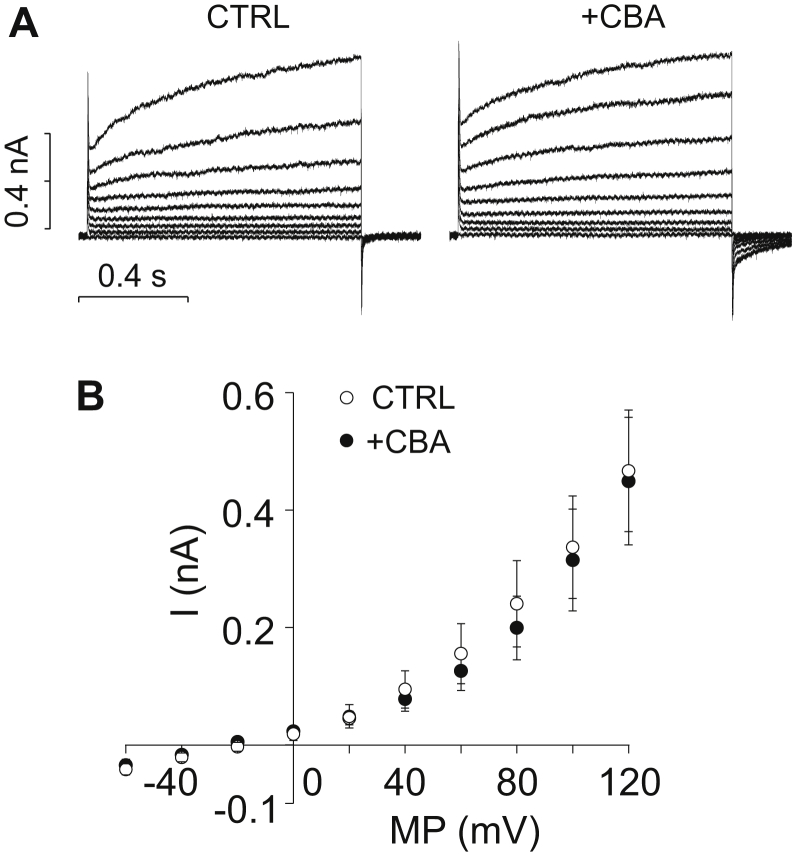
**CBA does not affect the Cl**^**−**^**current in pancreatic acinar cells.***A*, representative current traces of whole-cell currents of a cell under control conditions and during the application of 10 μM CBA. Step depolarizations were applied between −60 and +120 mV with 1 μM Ca^2+^ in the pipette solution. Averaged data are shown in panel *B* (n = 5). CBA, 4-chloro-2-[[2-(2-chlorophenoxy)acetyl]amino]benzoic acid.

In the next series of our experiments, functional expression of TRPM4 was tested using the voltage-clamp method in whole-cell configuration of the patch clamp technique. The ionic composition of the extracellular and intracellular solutions was designed specifically for the measurement of nonselective cation currents. To this end, much of Cl^−^ was replaced by glutamate in the recording medium, and Cs^+^ was added to the intracellular solution in order to prevent Cl^−^ and K^+^ currents, respectively. Averaged current traces, recorded in course of voltage ramp protocols applied between −60 and +120 mV, are shown in Figure 2, *A* and *B*. Under control conditions, a cation current appeared as a small inward background current displaying a slight voltage dependence at positive voltages. When the same cell was treated with cyclopiazonic acid (CPA), a specific inhibitor of SERCA (47), the current significantly increased. CPA is a common tool used to create elevated [Ca^2+^]_i_, as it inhibits Ca^2+^ reuptake and leaves resting ER Ca^2+^ leak uncompensated. As a result, CPA treatment raises [Ca^2+^]_i_ (35). The reason why we used CPA instead of secretagogue stimulation for this purpose is that CPA increases [Ca^2+^]_i_ while saving the PIP_2_ content of the plasma membrane, so it prevents rundown of TRPM4 current during the experiment (48). After reaching steady-state current, cells were treated with a solution containing CPA together with the widely used TRPM4 inhibitor 9-phenanthrol (9-ph; 100 μM) (49), which diminished the current (Fig. 2*A*). Unfortunately, 9-ph is not a fully selective inhibitor of TRPM4 as it is known to suppress also the Ca^2+^-dependent Cl^−^ current (50). This issue makes 9-ph problematic to apply with PACs, as both currents show significant depolarizing capacity in these cells. In order to overcome selectivity problems, a more specific TRMP4 inhibitor, 4-chloro-2-[[2-(2-chlorophenoxy)acetyl]amino]benzoic acid (CBA), was applied. About 10 μM of CBA inhibited the Ca^2+^-dependent cation current as potently as 100 μM 9-ph (Fig. 2*B*) (49) without affecting the Cl^−^ current (Fig. 3, *A* and *B*), indicating that CBA is an appropriate drug for selectively inhibiting TRPM4 even under the experimental conditions of live-cell Ca^2+^ imaging, when both cation and anion currents operate simultaneously in PACs.

Cation current was also measured in PACs isolated from animals in which the gene encoding the TRPM4 was disrupted (TRPM4 KO) (39), using the same experimental arrangement. Although, the mean current was somewhat higher comparing to control, it failed to increase during CPA treatment and was not sensitive to CBA either (Fig. 2*C*). These results strongly suggest that TRPM4 is functionally expressed in WT PACs in significant amounts.

**Figure 4:**
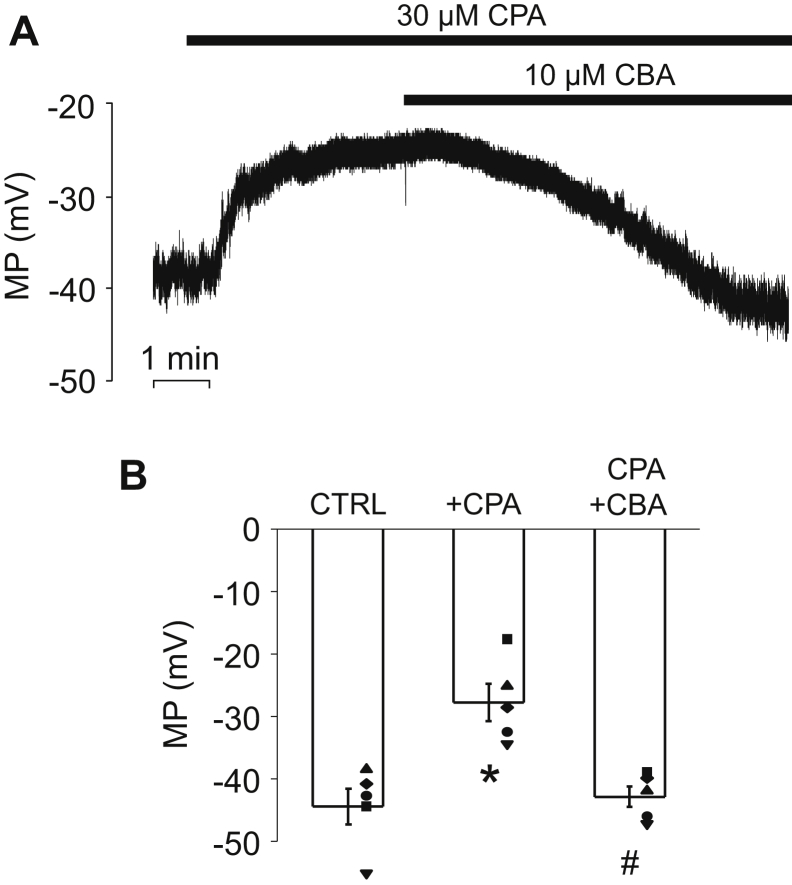
**Ca**^**2+**^**-dependent depolarization of pancreatic acinar cells relies on TRPM4 activity.***A*, representative membrane potential (MP) recording obtained under current-clamp conditions in control extracellular solution, during CPA (30 μM) and CPA + CBA (10 μM) treatment, respectively. Averaged membrane potentials recorded at different experimental conditions are shown in panel *B*. Mean values were compared with repeated-measures ANOVA, and pairwise comparisons between the indicated groups were carried out using paired-sample *t* tests with Bonferroni correction, *asterisks* indicate significant (*p* < 0.05) differences (n = 5). CBA, 4-chloro-2-[[2-(2-chlorophenoxy)acetyl]amino]benzoic acid; CPA, cyclopiazonic acid; TRPM4, transient receptor potential cation channel subfamily M member 4.

In order to determine the impact of TRPM4 current on membrane potential, perforated patch clamp experiments were performed using the current clamp technique. The membrane potential was −44.4 ± 2.9 mV under control conditions, and the membrane slowly depolarized to −27.7 ± 3 mV when 30 μM CPA was applied. The membrane potential returned close to the resting value (−42.9 ± 1.6 mV) when the perfusion solution was supplemented with 10 μM CBA (Fig. 4, *A* and *B*). Based on these results, we propose that PAC plasma membrane depolarizes in a Ca^2+^-dependent manner, involving the activation of TRPM4.

**Figure 5:**
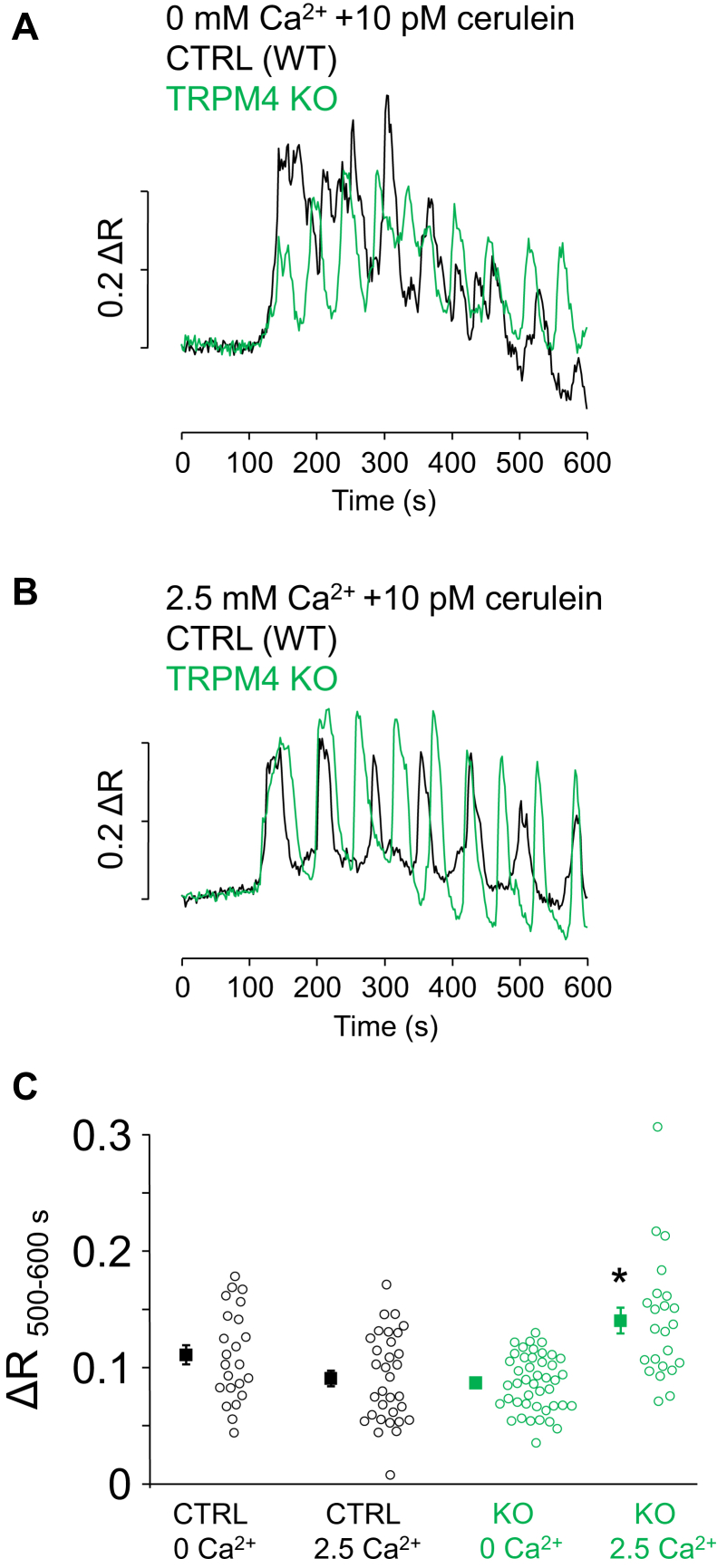
**TRPM4 affects the Ca**^**2+**^**signaling of mouse pancreatic acinar cells during CCK receptor stimulation.** Ratiometric fluorescent Ca^2+^ imaging was performed using Fura-8 AM-loaded pancreatic acinar cells isolated from WT or TRPM4 KO mice. Representative traces of fluorescence intensity ratios (Δ*R*) are displayed in *A* and *B*. Fluorescence was recorded in single cells of multiple individual cells of acinar cell clumps. Periodic fluctuations of [Ca^2+^]_i_ in response to 10 pM cerulein were recorded in extracellular saline containing 0 or 2.5 mM Ca^2+^ (*A* and *B*). Signal intensities of the spikes arising between 500 and 600 s were analyzed (Δ*R*_500–600 s_). Individual data (*circles*) and mean ± SE values (*square*) are shown in *C* (n = experiments/cells; n_CTRL 0 Ca2+_ = 3/23; n_CTRL 2.5 Ca2+_ = 4/33; n_KO 0 Ca2+_ = 6/44; n_KO 2.5 Ca2+_ = 3/23). *Asterisk* indicates significant differences (*p* < 0.05 one-way ANOVA, Bonferroni post hoc) as marked in the figure. CCK, cholecystokinin; TRPM4, transient receptor potential cation channel subfamily M member 4.

Furthermore, we hypothesized that the driving force for Ca^2+^ influx at these depolarized membrane potentials is low enough to significantly decrease Ca^2+^ entry. To test this hypothesis, ratiometric Ca^2+^ imaging was performed in clusters of PACs, which were exposed to long-term stimulation with low dose (10 pM) of cerulein, which is equivalent to a physiological stimulation with cholecystokinin (51). Parallel experiments were performed in Ca^2+^-containing and Ca^2+^-free solutions (when the source of Ca^2+^ can be only intracellular) using control and TRPM4 KO cells as well. Continuous application of 10 pM cerulein evoked periodic fluctuation (oscillation) of [Ca^2+^]_i_ in all groups (Fig. 5, *A* and *B*). Ca^2+^ spikes emerging between 8 and 10 min of cerulein treatment were analyzed because Ca^2+^ entry was expected to already contribute to the Ca^2+^ signaling by this time (21). While the average amplitude of Ca^2+^ spikes in control PACs was very similar in Ca^2+^-containing and Ca^2+^-free media, the value was markedly higher in Ca^2+^-containing medium in the case of TRPM4 KO preparations (Fig. 5*C*; ΔR_500–600 s_ values: CTRL 0 Ca^2+^: 0.111 ± 0.008; CTRL 2.5 Ca^2+^: 0.091 ± 0.007; TRPM4 KO 0 Ca^2+^: 0.087 ± 0.004; and TRPM4 KO 2.5 Ca^2+^: 0.14 ± 0.011). These data indicate that Ca^2+^ entry is significant after 8 min cerulein treatment in TRPM4 KO PACs but not in control cells. Importantly, the spike amplitudes in control and TRPM4 KO PACs were essentially the same in Ca^2+^-free saline solution, suggesting that the Ca^2+^ content of ER was similar at the end of cerulein treatment in both types of cells. Area under the curve (which is believed to be proportional to the sum of intracellular Ca^2+^) followed a same trend, but the differences were not different significantly (data not shown). Otherwise, the temporal characteristics of Ca^2+^ transients were not apparently different in control and TRPM4 KO PACs. In conclusion, the difference between control and KO PAC Ca^2+^ spikes in Ca^2+^-containing solution indicates that TRPM4 is likely involved in the negative feedback regulation of Ca^2+^ entry in PACs. Unfortunately, testing the role of TRPM4 using CBA in a similar experimental setting was not possible because of a strong off-target effect, that is, 30 μM CBA completely abolished Ca^2+^ oscillations in Ca^2+^-free bath solution (data not shown), suggesting that CBA inhibited Ca^2+^ release in PACs.

**Figure 6:**
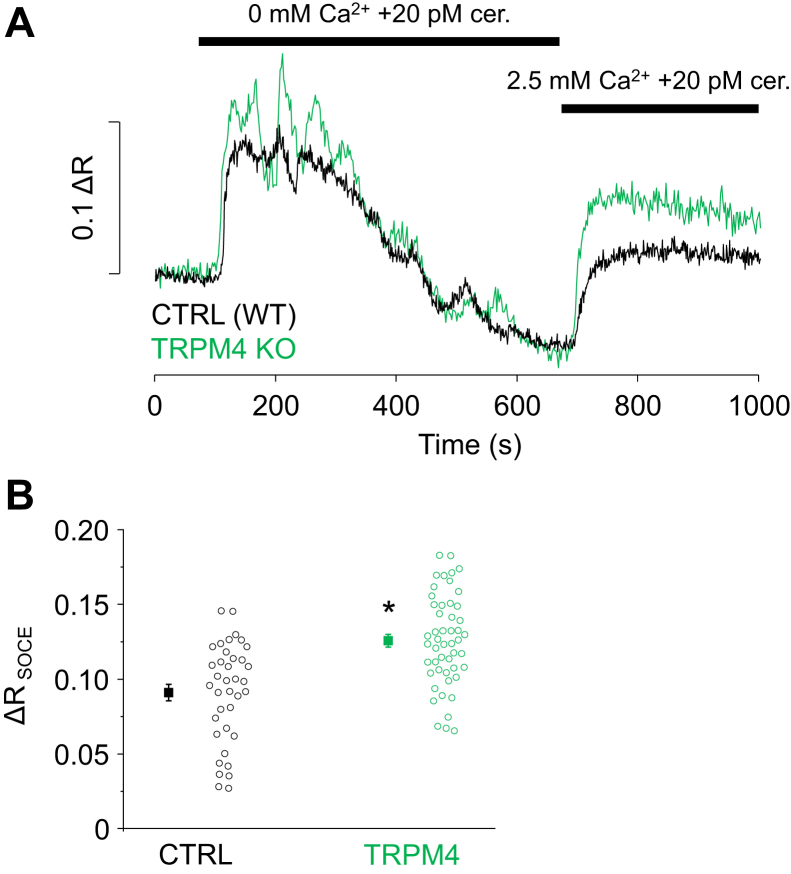
**TRPM4 activity regulates Ca**^**2+**^**entry during CCK receptor stimulation.** Ca^2+^ signals of WT and TRPM4 KO acinar cells treated with 20 pM cerulein in Ca^2+^-free extracellular solution and following a solution change to 2.5 mM Ca^2+^ (*black* and *green lines*, *A*). Statistics of the fluorescence amplitudes measured in 2.5 mM Ca^2+^ (*B*, Δ*R*_SOCE_; n = experiments/cells; n_CTRL_ = 6/37; n_TRPM4 KO_ = 7/50). *Asterisk* indicates significant differences (*p* < 0.05, Student's *t* test for independent samples). CCK, cholecystokinin; TRPM4, transient receptor potential cation channel subfamily M member 4.

The hypothesis that TRPM4 is involved in the negative feedback regulation of Ca^2+^ entry was further investigated in experiments designed to cause significant Ca^2+^ depletion from the ER in order to turn SOCE on. Therefore, PACs were stimulated with 20 pM cerulein for 10 min in Ca^2+^-free external solution. Cerulein treatment caused rather sustained Ca^2+^ signals with fluctuations of gradually decreasing amplitudes (Fig. 6*A*). This behavior is an obvious sign of ER depletion and Ca^2+^ unloading because of the activity of PMCA. Afterward, the solution was exchanged to a solution containing 2.5 mM Ca^2+^, which resulted in a tonic elevation of [Ca^2+^]_i_, attributed to the activation of SOCE (Fig. 6*A*). The amplitude of the SOCE-related fluorescence signal ratio (Δ*R*) was compared with those measured in TRPM4 KO PACs and found to be significantly higher in KO cells (Fig. 6*B*; Δ*R*_SOCE_ values: CTRL [WT]: 0.091 ± 0.005; TRPM4 KO: 0.126 ± 0.004).

**Figure 7:**
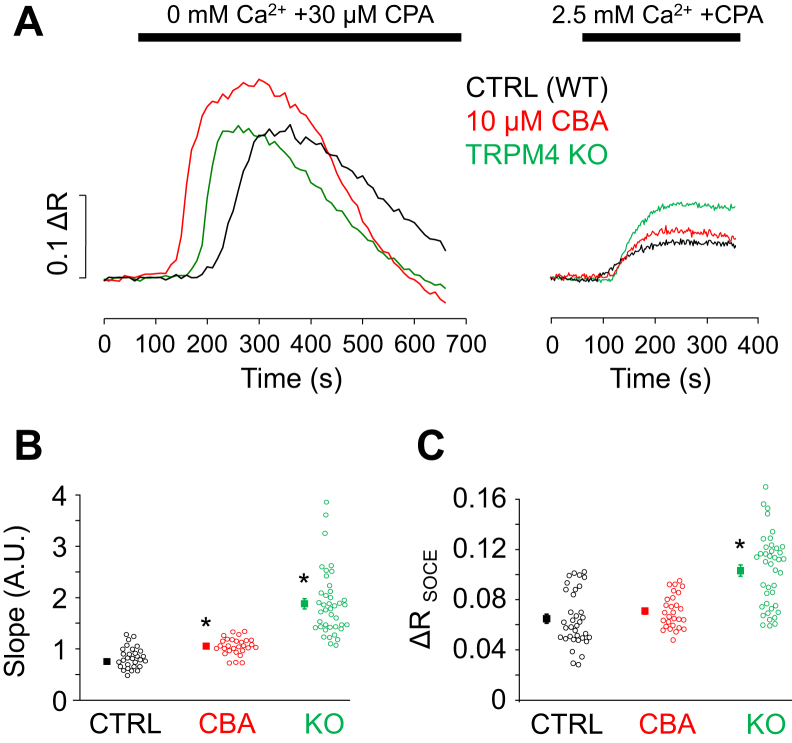
**TRPM4 activity regulates Ca**^**2+**^**entry evoked by CPA treatment.***A*, Ca^2+^ signals of control, CBA-treated, and TRPM4 KO acinar cells during 30 μM CPA application in Ca^2+^-free extracellular solution and following a solution change to 2.5 mM Ca^2+^. Statistics of the slope and amplitude of the signal (Δ*R*) recorded in 2.5 mM Ca^2+^ (*B* and *C*; n = experiments/cells; n_CTRL_ = 5/35; N_CBA_: 4/29; and n_KO_: 5/41). *Asterisks* indicate significant changes (*p* < 0.05, one-way ANOVA, Bonferroni post hoc) from control values. CPA, cyclopiazonic acid; TRPM4, transient receptor potential cation channel subfamily M member 4.

To verify these results, SOCE was more specifically investigated, by using CPA in order to cause ER depletion in a receptor-independent manner. This experimental approach avoids possible additional reactions that might interfere with the Ca^2+^-signaling machinery during secretagogue stimulations. About 30 μM of CPA was applied in Ca^2+^-free saline to induce Ca^2+^ leak from the ER (Fig. 7*A*). In the beginning of treatment, [Ca^2+^]_i_ increased, which was followed by a slow decrease, indicating that the ER depleted and Ca^2+^ was eliminated from the intracellular space by PMCA. The amplitude of Ca^2+^ signals was not significantly different between control, CBA-treated, and KO PACs (0.18 ± 0.02, 0.22 ± 0.01, and 0.21 ± 0.03, respectively). After reaching basal fluorescence values, the Ca^2+^-free solution was replaced by 2.5 mM Ca^2+^-containing solution, which resulted in a robust increase in [Ca^2+^]_i_ (Fig. 7*A*, *left panel*). Similar experiments were performed in the presence of CBA or using TRPM4 KO PACs. In these experiments, CBA was appropriate to use for the specific inhibition of TRPM4 because the ER was already depleted and the SERCA pump was inhibited; therefore, CBA could not affect Ca^2+^ release or the content of the ER. Analysis of Ca^2+^-influx–related alterations of fluorescence intensity ratios revealed that the slope and amplitude of the change of fluorescence (Δ*R*) was higher in TRPM4 KO PACs compared with control. In addition, CBA treatment significantly enhanced the rate of rise (but not the amplitude) of the fluorescence signal (Fig. 7, *B* and *C*) (slope, CTRL: 0.76 ± 0.04; CBA: 1.06 ± 0.03; KO: 1.88 ± 0.1 AU; Δ*R*_SOCE_: CTRL: 0.065 ± 0.004; CBA: 0.071 ± 0.002; KO: 0.103 ± 0.005). These data are in accordance with those presented in Figure 6 and support our hypothesis that TRPM4 is a negative feedback regulator of Ca^2+^ entry in mouse PACs.

## Discussion

In this study, we provide the first direct evidence that the TRPM4 current depolarizes PACs in a Ca^2+^-dependent manner and acts as a negative feedback regulator of Ca^2+^ entry under physiological conditions. However, our data may also have pathological implications. Ca^2+^ overload of PACs is believed to be the critical early pathological event, leading to premature intracellular zymogen activation, self-digestion, and eventually, acute pancreatitis (16, 17, 18). As SOCE is essential to develop sustained and pathological elevation of [Ca^2+^]_i_ and ORAI1 inhibitors were reported to mitigate the severity of acute pancreatitis, our data raise the possibility that TRPM4 plays a preventive role in the pathophysiology of Ca^2+^ signaling (34). This hypothesis should be further investigated using animal models of the disease. The translational and therapeutic potential of TRPM4 should be also evaluated.

Similar physiological functions of TRPM4 have been observed in T-lymphocytes and mast cells earlier. TRPM4 silencing transformed Ca^2+^ oscillations to sustained elevations of [Ca^2+^]_i_ and led to increased interleukin-2 production in Jurkat T cells, which is in accordance with the idea that TRPM4 reduces Ca^2+^ entry by depolarizing the plasma membrane and decreasing the driving force for Ca^2+^ influx (39).

Similarly, our results also imply that TRPM4 current suppresses Ca^2+^ entry by creating a depolarized membrane potential, where the driving force for Ca^2+^ entry is lower; however, an alternative mechanism is offered by Park *et al.* (52). They showed that TRPM4 physically interacts with TRPC3, a Ca^2+^ release–activated Ca^2+^ channel, in human embryonic kidney 293T cells, which results in reduction of channel activity. Since TRPC3 is highly expressed in PACs, this is a possible explanation for our results. In addition, although CBA was expected to block TRPM4, the compound failed to increase the amplitude of SOCE significantly, which might also be explained by the allosteric inhibition of TRPC3 by TRPM4. We presume that the inhibitory interaction between TRPM4 and TRPC3 is not affected by CBA; so TRPC3 is still inhibited by TRPM4 in the presence of CBA, which accounts for the unaltered SOCE amplitude. However, another reason might be that the TRPM4 inhibition is not complete in the applied concentration.

Apparently, the Cl^−^ current (mediated by the recently identified TMEM16a) also acts as a significant depolarizing current in PACs (53, 54, 55, 56, 57, 58), which raises the question why acinar cells express functionally redundant ionic currents. The Ca^2+^-dependent cation current was hypothesized earlier to have an additional function as a Na^+^ uptake channel of the basolateral membrane, which would supply a plausible transcellular Na^+^ transport mechanism with Na^+^. However, current measurements recorded at the equilibrium potential of Cl^−^ did not show increased cation current when [Ca^2+^]_i_ was elevated in the extreme apical region of the cell but only after [Ca^2+^]_i_ was increased in the whole intracellular space (59). Although in the lack of suitable TRPM4 antibodies for our immunofluorescence studies, we failed to demonstrate TRPM4 expression in PACs, this earlier study strongly suggests that Ca^2+^-activated cation channels are expressed only in the basal region of the plasma membrane. The results of Kasai and Augustine also imply that the apical membrane does not carry significant cation currents. Consequently, transepithelial fluid secretion is driven by a paracellular (not transcellular) Na^+^ transport, that is, TRPM4 does not participate in the fluid secretion process of PACs. Therefore, we conclude that TRPM4 functions only as a complementary depolarizing current, which is specifically localized in order to negatively regulate Ca^2+^ entry in the vicinity of Ca^2+^ release–activated Ca^2+^ channels.

## Experimental procedures

### PAC isolation

All experiments complied with the Hungarian Animal Welfare Act and the 2010/63/EU guideline of the European Union and were approved by the Animal Welfare Committee of the University of Debrecen.

Two types of mice were used in this study. The standard strain was C57Bl6, and we also used mice in which the gene encoding the TRPM4 was disrupted (TRPM4 KO). About 3- to 6-month-old mice of both genders were sacrificed by cervical dislocation, and the pancreas was removed immediately. Acinar cells were isolated as described earlier (60). The pancreas was injected with 100 U/ml collagenase P, 0.1 mg/ml trypsin inhibitor, and 2.5 mg/ml bovine serum albumin, dissolved in F12/Dulbecco's modified Eagle's medium (DMEM). The tissue was incubated in 5 ml of this solution in a shaking water bath at 37 °C for 25 min while continuously gassed with carbogen. The medium was replaced with fresh medium after 10 min. The tissue was dissociated by trituration performed by 4 to 6 cycles of pipetting through a 10-ml serological pipette, then filtered through a 150 μm mesh. Cells were layered on the top of 2 × 5 ml F12/DMEM, containing 400 mg/ml bovine serum albumin and collected by gentle centrifugation. The pellet was washed in 2 ml DMEM and collected by slow centrifugation. Acinar cell clumps were gently resuspended in DMEM and kept at room temperature until use in Ca^2+^ imaging experiments.

In order to gain single acinar cells for electrophysiological measurements, the resulting acinar cell clumps were subjected to an additional digesting step in Ca^2+^ and Mg^2+^-free PBS containing 100 U/ml collagenase P for 10 min. Thereafter, cells were dissociated with pipetting using a 5-ml serological pipette.

### Intracellular Ca^2+^ imaging

Acinar cell clumps were loaded with 2 μM Fura-8 AM (AAT Bioquest) Ca^2+^ sensitive dye for 30 min at room temperature. Acinar cells were plated on glass-bottom dishes (Bioptechs) and allowed to attach to the bottom. Cells were perfused with Tyrode's solution containing (in millimolar): 140 NaCl, 5 KCl, 2 MgCl_2_, 2.5 CaCl_2_, and 10 Hepes, and pH 7.38. Some experiments were performed with a Ca^2+^-free Tyrode's solution, which contained 0.5 EGTA, but no CaCl_2_. Ratiometric measurement of Fura-8 fluorescence was performed at room temperature using a Zeiss Axiovert 135 microscope equipped with a 40× Fluor (1.3 numerical aperture) objective. Fura-8 was excited at 360 and 405 nm at 1 Hz using an light-emitting diode light source (FuraLED; Cairn Research Ltd), and the emitted light was passed through a 520-nm longpass filter and collected using a Qimaging Retiga R3 charge-coupled device camera. The imaging hardware setup was controlled by Micromanager software (an open source software program) (61, 62) through an interface. Fluorescence values were determined using ImageJ (National Institutes of Health) software with Fiji plugins. Fluorescence ratios of emissions elicited by excitations at 360 and 405 nm were calculated after background subtraction in single cells. Changes of ratios (Δ*R*) were determined for each cell, averaged, and presented as mean ± SEM.

### Electrophysiological recordings

Whole-cell currents were recorded at room temperature using an Axopatch 200B amplifier and a Digidata 1320A digitizer (Molecular Devices) at a 50-kHz sampling rate and filtered online at 5 kHz using a low-pass Bessel filter. Patch pipettes of 5 to 7 MΩ resistance were pulled from borosilicate glass capillaries (Warner Instruments). In TRPM4 current measurements, pipettes were filled with a solution containing (in millimolar): 144 Cs-glutamate, 1 MgCl_2_, 0.1 EGTA, 0.0486 CaCl_2_ (100 nM ionized Ca^2+^), 3 K-ATP, 10 Hepes, at pH 7.3. The external solution contained (in millimolar): 140 sodium glutamate, 4 CsCl, 2 MgCl_2_, 10 Hepes, at pH 7.4.

In Cl^−^ current measurements, the pipette solution contained (in millimolar): 140 *N*-methyl-d-glucamine chloride, 1 MgCl_2_, 1.72 CaCl_2_, 2 EGTA (1 μM ionized Ca^2+^), at pH 7.2. The extracellular solution contained (in millimolar): 140 *N*-methyl-d-glucamine chloride, 1 MgCl_2_, 5 glucose, 10 Hepes, at pH 7.3. All ingredients of the solutions were purchased from Sigma–Aldrich (Merck).

TRPM4 current was measured using a ramp voltage protocol applied from −100 to +120 mV, whereas Cl^−^ current was recorded during 1 s long step depolarizations applied between −60 and +120 mV. At least 70% of the series resistance was compensated in these measurements.

Membrane potential was measured under current-clamp condition using the perforated patch clamp technique. The cells were bathed in Tyrode's solution, whereas the tip of the pipette was filled with a solution containing (in millimolar): 85 potassium glutamate, 45 KCl, 15 NaCl, 2 MgCl_2_, 0.1 EGTA, 0.0486 CaCl_2_ (100 nM ionized Ca^2+^), 10 Hepes at pH 7.3. The pipette was back-filled with the same solution, supplemented with 300 μg/ml amphotericin B.

### RNA isolation, RT, and quantitative real-time PCR

qPCR was performed on a Roche LightCycler 480 System (Roche) using the 5′ nuclease assay (63). Total RNA was isolated using TRIzol (Life Technologies Hungary Ltd), DNase treatment was performed according to the manufacturer's protocol, and then 1 μg of total RNA was reverse-transcribed into complementary DNA using High-Capacity cDNA Kit from Life Technologies Hungary Ltd. PCR amplification was performed using the TaqMan Gene Expression Assays (assay IDs: Mm01175211_m1 for RYR1, Mm00465877_m1 for RYR2, Mm01328421_m1 for RYR3, Mm00439907_m1 for inositol 1,4,5-trisphosphate receptor (ITPR) type 1, Mm00444937_m1 for ITPR type 2, Mm01306070_m1 for ITPR type 3, Mm00613173_m1 for TRPM4, Mm01129032_m1 for TRPM5, and Mm00444690_m1 for TRPC3) and the TaqMan universal PCR master mix protocol (Applied Biosystems). As internal control, transcripts of the housekeeping gene (GAPDH; assay ID: Mm99999915_g1) were determined. The amount of the transcripts was normalized to the housekeeping gene using the ΔCT method.

### Chemicals

Fura-8 AM was purchased from AAT Bioquest. CBA was from Tocris Bioscience (Bio-Techne Corporation). All other chemicals (including collagenase P, 9-ph, CPA, and cerulein) were obtained from Sigma–Aldrich (Merck).

### Statistical analysis

Analysis was made in Origin 7.0 (Microcal Software) or in Microsoft Excel. Data are presented as the average of cells obtained from at least three independent experiments and at least three animals. Averages are expressed as mean ± SEM. Statistical analysis was performed by using Student's *t* test or one-way ANOVA with Bonferroni post-test. Related samples were analyzed using repeated-measures ANOVA, and pairwise comparisons were carried out with Bonferroni-corrected paired sample *t* test. Differences were considered significant when *p* was less than 0.05.

The number of experiments (n) denotes the number of experimental repeats/total number of cells in the case of Ca^2+^ imaging and the number of cells in patch clamp measurements as indicated in the legends to the figures.

## Data availability

All data are contained within the article and available upon request.

## Conflict of interest

The authors declare that they have no conflicts of interest with the contents of this article.
